# Changes of lung tumour volume on CT - prediction of the reliability of assessments

**DOI:** 10.1186/s40644-015-0052-2

**Published:** 2015-10-31

**Authors:** Hubert Beaumont, Simon Souchet, Jean Marc Labatte, Antoine Iannessi, Anthony William Tolcher

**Affiliations:** MEDIAN Technologies - Sciences, 1800 route des crêtes Les Deux Arcs Bat B, Valbonne, 06560 France; Université d’Angers - Mathematics, Angers, 49000 France; Centre Anticancer Antoine Lacassagne - Radiology, Nice, 06100 France; START - South Texas Accelerated Research Therapeutics, LLC - Clinical Research, San Antonio, TX USA

**Keywords:** Patient monitoring, Medical oncology, Drug response biomarkers, Reproducibility of results, Decision support techniques

## Abstract

**Background:**

For oncological evaluations, quantitative radiology gives clinicians significant insight into patients’ response to therapy. In regard to the Response Evaluation Criteria in Solid Tumours (RECIST), the classification of disease evolution partly consists in applying thresholds to the measurement of the relative change of tumour. In the case of tumour volumetry, response thresholds have not yet been established. This study proposes and validates a model for calculating thresholds for the detection of minimal tumour change when using the volume of pulmonary lesions on CT as imaging biomarker.

**Methods:**

Our work is based on the reliability analysis of tumour volume measurements documented by the Quantitative Imaging Biomarker Alliance. Statistics of measurements were entered into a multi-parametric mathematical model of the relative changes derived from the Geary-Hinkley transformation. The consistency of the model was tested by comparing modelled thresholds against Monte Carlo simulations of tumour volume measurements with additive random error. The model has been validated by repeating measurements on real patient follow ups.

**Results:**

For unchanged tumour volume, relying on a normal distribution of error, the agreement between model and simulations featured a type I error of 5.25 %. Thus, we established that a threshold of 35 % of volume reduction corresponds to a partial response (PR) and a 55 % volume increase corresponds to progressive disease (PD). Changes between −35 and +55 % are categorized as stable disease (SD). Tested on real clinical data, 97.1 % [95.7; 98.0] of assessments fall into the range of variability predicted by our model of confidence interval.

**Conclusions:**

Our study indicates that the Geary Hinkley model, using published statistics, is appropriate to predict response thresholds for the volume of pulmonary lesions on CT.

## Background

Imaging is essential in the diagnosis and management of patients with cancer, both in clinical practice and clinical research. Among the available imaging modalities, computed tomography (CT) is largely used in oncology for the initial evaluation of lesions and to characterize changes in tumour measurements [1–3].

Visual evaluations of CT images are generally sufficient to make general assessments when clinical examinations or changes are obvious. Subjective assessments are limited, however, when changes are modest or confounded by inter- and intra-observer variability. A recent publication suggests that the early adoption of reproducible and reliable quantitative imaging biomarkers (QIB) may prevent large and costly clinical phase III studies based on flawed response data from earlier studies [4, 5].

The Response Evaluation Criteria in Solid Tumours (RECIST) [6] is a standard that, in part, relies on a simplified approach of the measurement of lesions in considering their longest axial diameters (LD) and the categorization of their changes between response, progression and stability. Despite the interest of such a simple approach, several limitations have been reported [7] as the concerns about reliability of measurements and its inadequacy in disease settings in which tumour measurements are difficult or uninformative.

In addition, response categories as defined by RECIST may fail to capture the activity of novel therapies [8, 9] by categorising as unfavourable stable disease situations that, some authors believe, may indicate drug effectiveness for targeted therapies [10]. Volumetric measurements are, by nature, able to capture tumour changes along the z-axis, unlike uni- and bi- dimensional measurements where tumour changes are measured on a transverse image plane. With this added information, several groups shown that, for pulmonary disease, volumetric measurements could make assessments more sensitive and more specific to disease change over time [11–14].

Before tumour volumetry can be effectively adopted, the biomarker must be qualified in determining the foremost factors affecting the reliability of assessments and the limitations set by unstandardized practice [15]. For this purpose, the Quantitative Imaging Biomarker Alliance (QIBA) [16] has enabled the creation of an active community to work at qualifying the volume of tumour on CT as a biomarker and at standardizing procedures [17].

Amongst other requirements, the qualification process of QIBs must estimate the reliability in assessing changes in the tumour burden. The estimate of reliability allows for classification of partial responses (PR), stable disease (SD) or progressive disease (PD) based on decisional thresholds supposed to reflect change in the health status of patients. But classification of health status remains challenging, and, unfortunately, there are no experiments reporting repeated measurements of change for tumours truly changing over time with a known volume increase or decrease.

Because it is not feasible to repeat biopsy in patients to confirm true lesion change, the alternative is usually to characterize the variability of the dual situation known as “non-change” condition. Characterization can be carried out relying on repeated measurements of uncorrelated lesions and/or processing test-retest data. However, relying on these methodologies [18], the qualification of QIBs remains incomplete because, to date, no method has been suggested to characterize “change” conditions based on “non-change” outcomes.

In the present study, we adopted a value for the variability of repeated measurements of uncorrelated lesions as published by other groups. We use this value of variability as a starting hypothesis to run a mathematical model able to predict the decisional thresholds classifying changes in tumour volume. Simulation studies and validation on real clinical data confirmed the effectiveness of the method for the monitoring of the volume of pulmonary lesions using CT.

## Methods

### Starting hypothesis

A fundamental input needed by our mathematical model is the magnitude of the variability of volumetric assessment. We hypothesized this value based on works published by other groups.

We analysed results and conclusions from different sources, mainly from the data released by the QIBA Volume CT group, which we found to be the best documented, particularly regarding the standard deviation of repeated measurements [17].

We reviewed additional publications to confirm the standard deviation value, notably using test-retest data where patients are scanned twice within a short period of time, guaranteeing that no clinical change happened. Again, studies showed that a conservative value of the standard deviation of 15 % can be considered when lesions are not too small [19].

### Statistical model

Let *X*_*1*_ be the volume measurement at a given time point and *X*_*2*_ the measurement at a second time point. Inference about the relative difference, as used by RECIST, is equivalent to the inference about the ratio of two volumes because *(X*_*2*_*-X*_*1*_*)/X*_*1*_ *= X*_*2*_*/X*_*1*_*-*1.

Our first goal was to draw confidence interval attached to such ratio in its most general form then restricted to our specific application.

Inference about a ratio of parameters is a well-known problem in statistics. *X*_*1*_ and *X*_*2*_ are obviously considered as two random variables with expected values μ_i_, standard deviation σ_i_, coefficient of variation *c*_*i*_*, i* = 1,2 respectively, and the coefficient of correlation ρ. Assuming that (*X*_*1*_, *X*_*2*_) is approximately distributed as a bivariate normal random variable and noting the ratio *W* = *X*_*2*_/*X*_*1*_, the Geary-Hinkley transformation, *Z,* of *W* [20] is:

$$ Z=\left(W{\mu}_1-{\mu}_2\right)/\sqrt{{\displaystyle {\sigma}_2^2}-2W\;\rho {\sigma}_1{\sigma}_2+{W}^2{\displaystyle {\sigma}_1^2}} $$

The new variable, *Z,* may be approximated by the standard normal distribution under the suitable conditions: *X*_*1*_ > 0, *X*_*2*_ > 0, *c*_*1*_ < 0.39 and *c*_*2*_ > 0.005 [21]. These conditions are verified in this study with *c*_*1*_ and *c*_*2*_ reported around 0.15 by QIBA. Correct lower and upper limits for the confidence interval of μ_2_/μ_1_ could then be deduced from this transformation with [22]:

$$ Lower=\frac{X_2}{X_1}\;\frac{1-{t}^2\;\rho {c}_1{c}_2-t\;\sqrt{c_2^2-2\rho {c}_1{c}_2+{c}_1^2-{t}^2{c}_1^2{c}_2^2\left(1-{\rho}^2\right)}}{1-{t}^2{c}_2^2} $$

$$ Upper=\frac{X_2}{X_1}\;\frac{1-{t}^2\rho {c}_1{c}_2+t\;\sqrt{c_2^2-2\rho {c}_1{c}_2+{c}_1^2-{t}^2{c}_1^2{c}_2^2\left(1-{\rho}^2\right)}}{1-{t}^2{c}_2^2} $$

where *t* is the appropriate quantile of the standard normal distribution.

In addition to the calculation of the confidence interval associated to ratio evaluation, *p*-value and statistical power could be deduced from the data (see Appendix).

### Simulation studies

The statistical model is based upon several simplified hypotheses: normal distribution of *X*_*1*_ and *X*_*2*_, no correlation between *measurement errors* and a constant coefficient of variation. We tested the robustness of the statistical model to these hypotheses with Monte-Carlo simulations. We compared the theoretical *p*-value and statistical power drawn from the Geary-Hinkley transformation against the *p*-value and statistical power computed from simulations relying on several different model of error and distributions.

Three error models were simulated: independent normally distributed random variables, independent log-normally distributed random variables, and empirical uniform distribution. When considering constant coefficient of variation as *c*_*1*_ = *c*_*2*_ = *k*, standard error was calculated as: k * μ_1_ = (0,3/1,96) * μ_ι_, according to QIBA report [17] for the two first error models.

For each error model, we simulated four ratios between time points: No change, the thresholds we found (−35 %; +55 %) and the threshold proposed by QIBA (+30 %). For unchanged volume, the rejection rate corresponds to the type I error of the test. For the three threshold values, the rejection rate corresponds to the statistical power of the test.

For each error model and change, we generated 10,000 samples from two independent distributions with expected values μ_1_ and μ_2_. The percentage of rejection was calculated as the percentage of observation which is not in the 95 % confidence interval. The statistical power of the test is thus the percentage of observations rejected. Statistical analyses and simulations were conducted using the “R” software package [23].

### Testing on real clinical data

We used image patients that were originally provided by Merck & Co Inc to the QIBA CT Technical Committee On Volumetry [12, 15]. A sample of 10 patients were retrospectively selected from a multi-center study if they had 5 or more analysable CT scans after their baseline scan. Patients have stage IIIB or stage IV non-small cell lung cancer (NSCLC).

CT acquisition protocols were different at each time point for 70 % of the patients.

CT scan slice thickness ranged from 1 mm to 7 mm (8.5 %) with most of scans performed with a thickness of 5 mm (87 %). Most of the scans were acquired with a 120kVp tube voltage.

Data were acquired from 10 different scanners, from three different manufacturers (Siemens, General Electric and Toshiba). Voxel size ranged from 0.5 mm to 1 mm.

Median, minimum and maximum size of LD was 40 mm, 11 mm and 117 mm respectively.

A total of 426 lesion measurements were then performed on 71 time-points according to a sample size that was defined by previous studies [24, 25]. On each scan, a single lung target lesion was preselected by an expert radiologist to ensure comparable lesions assessment across readers. The expert radiologist defined a mark attached to each lesions enrolled in the dataset. In order to minimize segmentation bias, marks were defined to lie within the tumour margin not on the maximum cross-sectional area and readers were asked to delete the mark after lesion identification and before segmenting.

Three experienced radiologists and three experienced image scientists with expertise in medical image processing and analysis were involved in the measurements. For each tumour and at each time point, readers segmented lesion contours for measurement of tumour volume, so that each of the six readers assessed all the dataset.

Volume segmentation was performed sequentially without concomitant display of prior segmentations or results but in chronological order. The segmentation process was initiated on the slice where tumour appeared the largest.

Volume assessments were based on a semi-automatic tool and a manual tool able to refine by modifying previous segmentations. The same software was used by all readers (LMS, Median Technologies, Valbonne, France).

Based on the assessment of tumour volume, we computed the relative change with respect to the measurement performed at the first time point which was considered as the reference. We performed a two-way evaluation by considering either baseline or nadir measurement as reference time point to balance our dataset as 68 responding and 67 progressive responses by readers. We have not investigated the possible bias introduced by our design when considering nadir as reference, tumours volume at nadir being, in average, smaller than at baseline.

Relative changes measured by each reader was reported against the average relative change measured by the group. The scattering of readers assessment at a given time point was tested to fall into the confidence interval predicted by our model.

Finally, we used the thresholds we found for the monitoring of volume changes to extrapolate thresholds applicable to the monitoring of effective diameter changes. We adopted the definition of the effective diameter of tumours as the diameter computed from a sphere whose volume is the same as the measured nodule volume.

## Results

### Measurements

The mean volume ranged from 29.466 cm^3^ to 30.719 cm^3^ with volume ranges from 0.195 cm^3^ to 380.976 cm^3^. Majority of measurements were comprised between 1.0 and 100.0 cm^3^.

### Confidence interval for volume

We relied on the simplest hypotheses. First, the two measurements were considered as normally distributed and independent random variables. Second, variances were assumed to be proportional to the means, based on the QIBA conclusion.

In the case where *ρ* = 0, *c*_1_ = *c*_2_ = *k* the confidence interval simplifies itself in

$$ Lower=\frac{X_2}{X_1}\;\frac{1-t\;\sqrt{2{k}^2-{t}^2{k}^2}}{1-{t}^2{k}^2} $$

$$ Upper=\frac{X_2}{X_1}\;\frac{1+t\;\sqrt{2{k}^2-{t}^2{k}^2}}{1-{t}^2{k}^2} $$

The thresholds of the 95 % confidence interval to test the null hypothesis that the ratio is one, i.e., no change of tumour volume, are for the lower threshold,

$$ \frac{1-1.96\;\sqrt{2*{\left(0.3/1.96\right)}^2-{1.96}^2\;{\left(0.3/1.96\right)}^4}}{1-{1.96}^2\;{\left(0.3/1.96\right)}^2}\approx 0.65 $$

a decrease of 35 %, and for the upper threshold,

$$ \frac{1-1.96\;\sqrt{2*{\left(0.3/1.96\right)}^2+{1.96}^2{\left(0.3/1.96\right)}^4}}{1-{1.96}^2{\left(0.3/1.96\right)}^2}\approx 1.55, $$

an increase of 55 %.

**Fig. 1:**
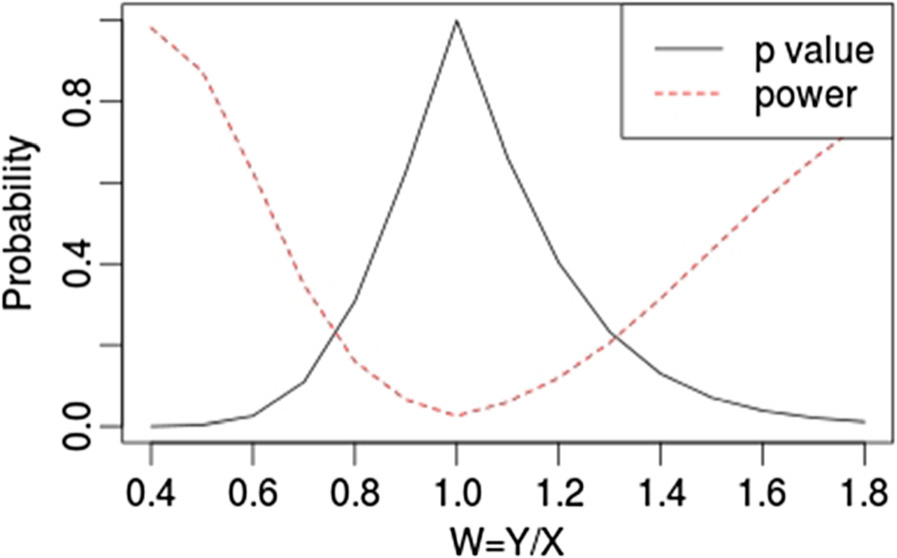
According to our Geary-Hinkley model, solid dark line simulates the p-value and dashed red line simulates the statistical power for a ratio of random variable ranging from 0.4 to 1.8

Figure 1 describes the *p*-value and statistical power in relation with the observed ratio *X*_*2*_/*X*_*1*_. The power curve displays the statistical power, which is the probability of declaring response as a function of the known volume ratio according to Equation 2. For a volume decrease of 50 %, 35 % and 20 %, the statistical power is respectively 87.5 %, 50 % and 16 %. For a volume increase of 30 %, 55 % and 80 %, the statistical power is respectively 20.6 %, 50 % and 75 %.

### Simulation studies

The performance of the proposed thresholds was assessed through simulation studies. These simulations evaluate the coverage probabilities of the confidence interval and the type I error rates and powers under several change and hypotheses of error model.

**Fig. 2:**
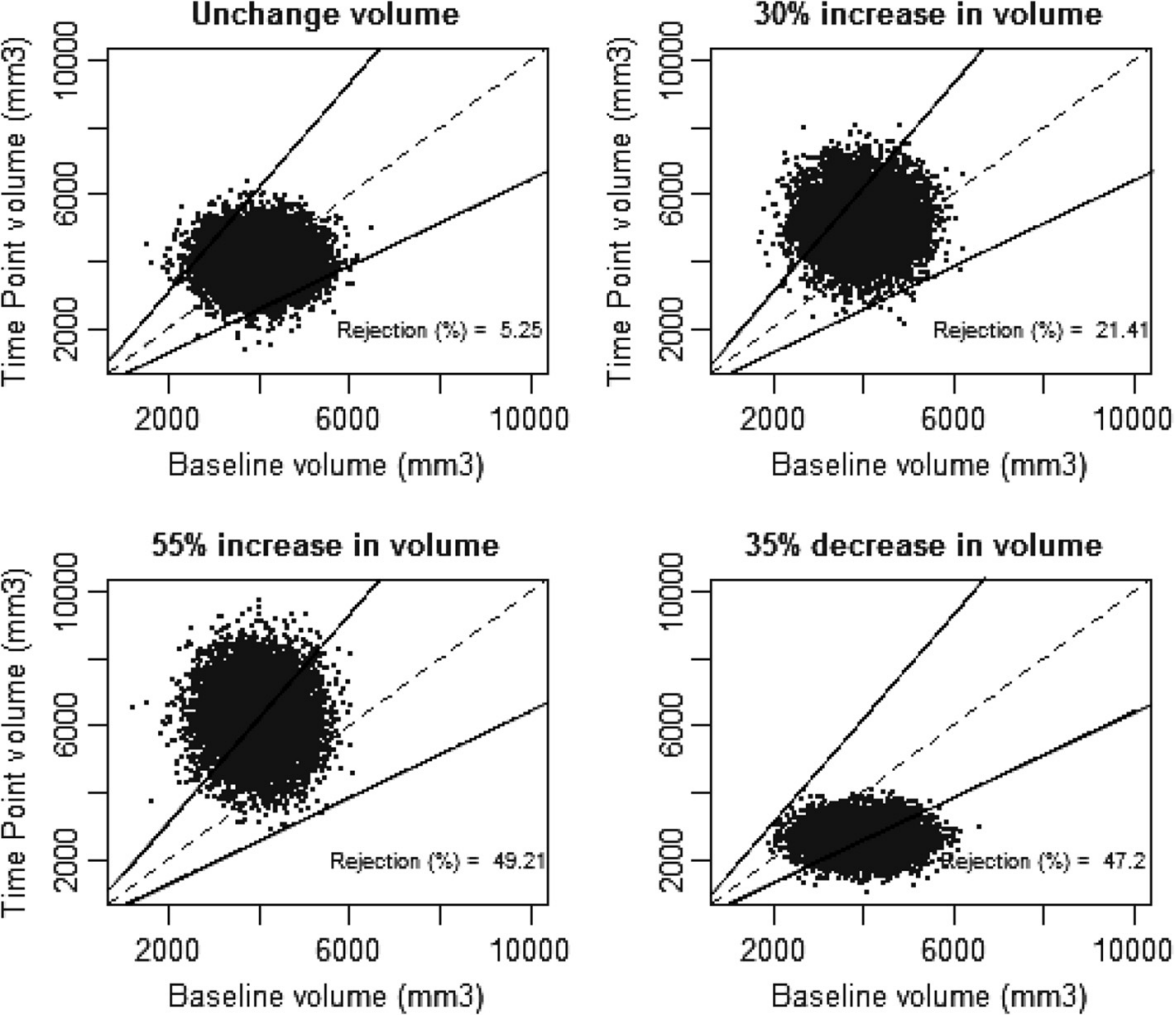
Simulation of couple of normally distributed and independent random variables. Horizontal axis is the value of the first random variable as the value of lesion volume at baseline, vertical axis is the value of the second random variable as the value of lesion volume at a later Time Point. Dashed first diagonal is the line of equivalence (No change), solid line correspond to thresholds as -35 %; +55 % change. a) Top left: Simulation of random variable having same value (Rejection=5.25 %). b) Top right: Simulation of a 30 % increase of the random variable. (Rejection=21.41 %). c) Bottom left: Simulation of a 55 % increase of the random variable. (Rejection=49.21 %). d) Bottom right: Simulation of 35 % decrease of the random variable. (Rejection=47.2 %)

**Fig. 3:**
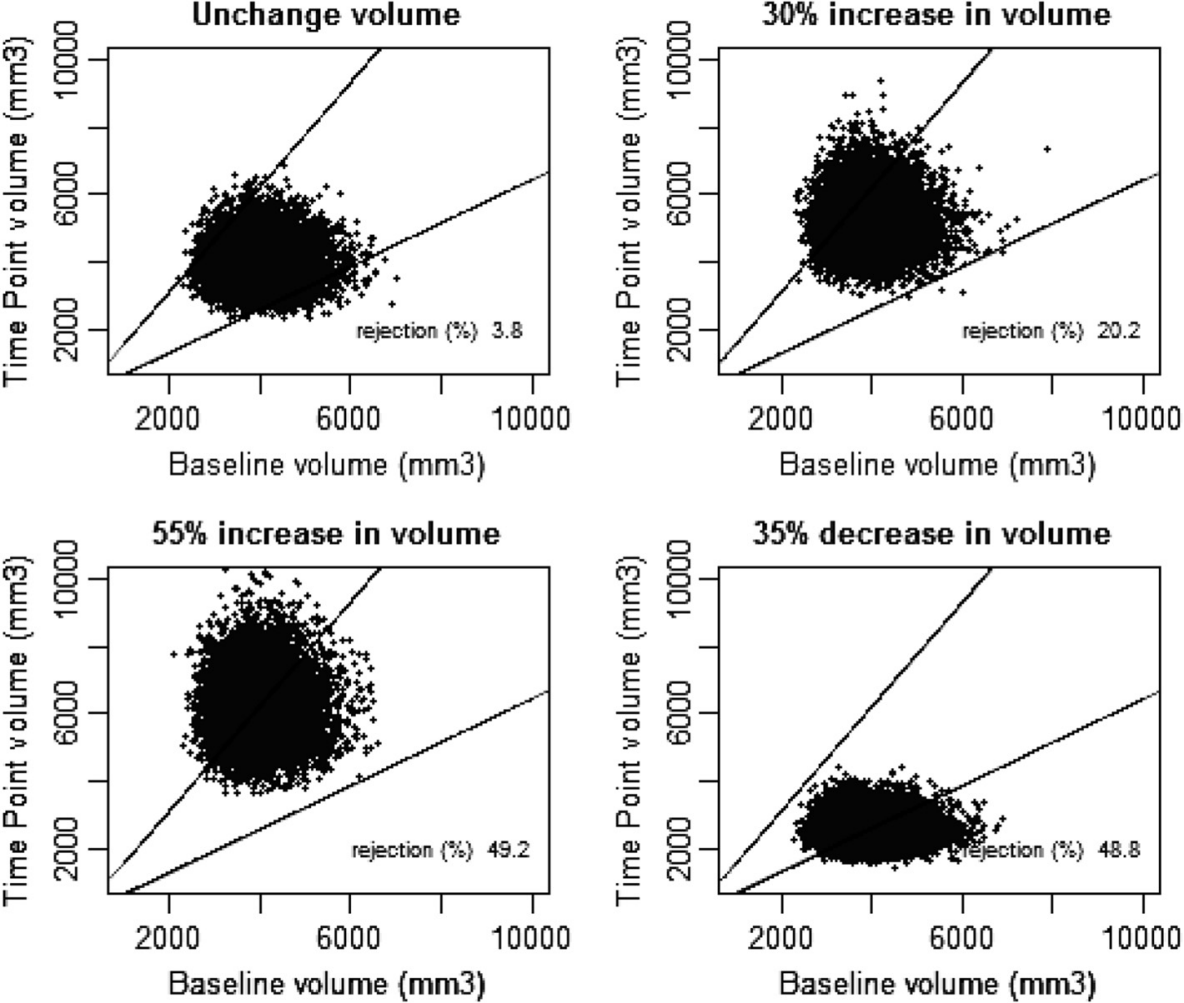
Simulation studies with lognormal distributed and independent random variables. Horizontal axis is the value of the first random variable as the value of lesion volume at baseline, vertical axis is the value of the second random variable as the value of lesion volume at a later Time Point. Dashed first diagonal is the line of equivalence (No change), solid line correspond to thresholds as -35 %; +55 % change. a) Top left: Simulation of random variable having same value (Rejection=3.8 %). b) Top right: Simulation of a 30 % increase of the random variable. (Rejection=20.2 %). c) Bottom left: Simulation of a 55 % increase of the random variable. (Rejection=49.2 %). d) Bottom right: Simulation of 35 % decrease of the random variable. (Rejection=48.8 %)

**Fig. 4:**
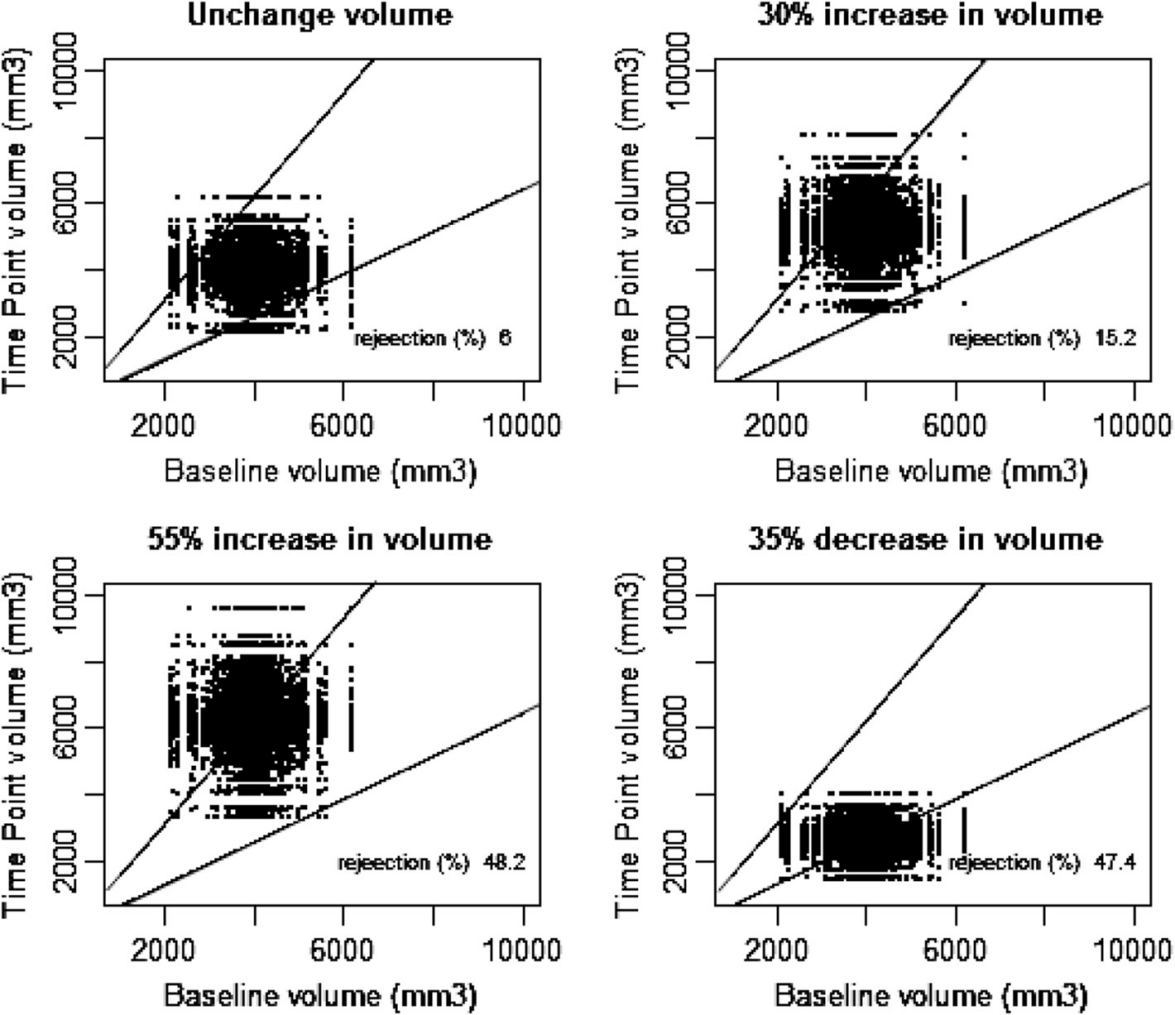
Simulation studies with empirical model error. Horizontal axis is the value of the first random variable as the value of lesion volume at baseline, vertical axis is the value of the second random variable as the value of lesion volume at a later Time Point. Dashed first diagonal is the line of equivalence (No change), solid line correspond to thresholds as -35 %; +55 % change. a) Top left: Simulation of random variable having same value (Rejection=6 %). b) Top right: Simulation of a 30 % increase of the random variable. (Rejection=15.2 %). c) Bottom left: Simulation of a 55 % increase of the random variable. (Rejection=48.2 %). d) Bottom right: Simulation of 35 % decrease of the random variable. (Rejection=47.4 %)

For unchanged volume simulation (Figs. 2a, 3a, 4a), the range of *p*-value was between 3.8 % (lognormal model) and 6 % (empirical error), near the 5 % expected value. For an increase of 30 % of tumour volume (Fig. 2b, 3b, 4b), the range of statistical power was between 15 % (empirical error) and 21.4 % (normal error). The theoretical expected value is 20.6 %. For an increase of 55 % of nodule volume (upper threshold value) (Fig. 2c, 3c, 4c), the range of statistical power was between 48.2 and 49 %. The theoretical expected value is 50 %. For a decrease of 30 % of nodule volume (lower threshold value) (Fig. 2d, 3d, 4d), the range of statistical power is 47.2 to 48.4 %. The theoretical value is 50 %.

### Test on real clinical data

**Fig. 5:**
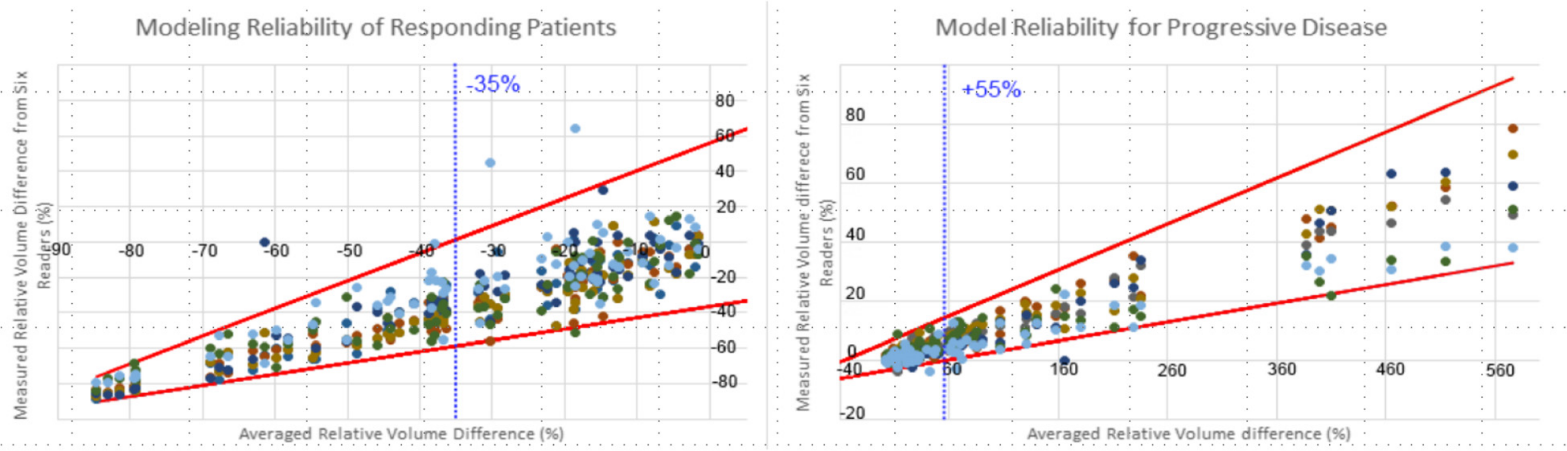
Validation of the model with real patient follow-up data. 6 Experienced readers measured volumetric tumour change involving 10 patients and 71 time points. A two-way evaluation was performed in considering either baseline or nadir measurement as reference to balance the assessments between 68 decreasing and 67 increasing changes. Horizontal axis reports the average response assessed by all readers. Vertical axis reports change assessment for each reader, each of them identified by a spot of different colour. Red lines represent the confidence interval predicted by Geary-Hinckley model. 97.1 % of readers’ assessment fall into predicted range of error. Left to -35 % dotted blue line are represented responding assessments, right to +55 % dotted blue line are represented progressive assessment

**Fig. 6:**
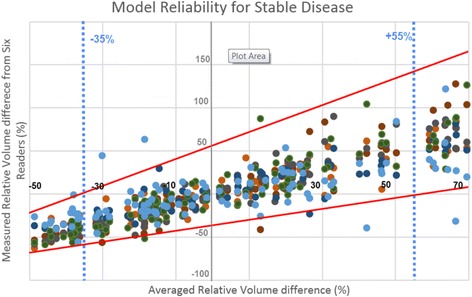
Validation of the model with real patient follow-up data. 6 Experienced readers measured volumetric tumour change involving 10 patients and 71 time points. A two-way evaluation was performed in considering either baseline or nadir measurement as reference to balance the assessments between 68 decreasing and 67 increasing changes. Horizontal axis reports the average response assessed by all readers. Vertical axis reports change assessment for each reader, each of them identified by a spot of different colour. Red lines represent the confidence interval predicted by Geary-Hinckley model. 97.1 % of readers’ assessment fall into predicted range of error. Between -35 % and +55 % blue dotted lines are represented stable assessments

According to our model of confidence interval, 97.1 % [95.7; 98.0] of assessments fall into predicted range of variability as depicted on Figs. 5 and 6. The model is robust in assessing response, stable or progressive disease. Significant association was found between assessing progression and reliability of each side of the CI (p = 0.02).

### Extrapolation to effective diameter

Based on the thresholds previously established for volume (−35 %; +55 %), we found that thresholds of −13 %; 16 % are applicable to the monitoring of effective diameters. We can see that the thresholds applicable to effective diameter define a narrower region for stable disease than the −30 %; 20 % applicable to one-dimensional measurements according to RECIST [6].

## Discussion

The design of a usable response criteria requires the establishment of a set of decisional thresholds that is able to detect the minimum meaningful change in volume. To be realistic, sensitivity of thresholds must take into account the variability introduced by imaging, measurements and anatomy. Several groups studied the volumetric minimum meaningful change according to different approaches. Analysing non-change conditions, Wang and al. reported that, when repeating segmentations, only 2 % of the 4225 studied nodules had proportional volume differences of more or less than 25 % [26]. When variability encompasses anatomical changes, test-retest studies suggested that proportional volume differences outside the range −12.1 to +13.4 % [27], or for smaller lesions, −21.2 %; 23.8 % [28], could be considered as a true change with a 5 % risk error. For changing conditions, when sensitivity is associated with the threshold, a 24.9 % relative decrease classified 90 % of tumours with a mutation as responders and 89 % of tumours without a mutation as non-responders [29]. Extrapolating RECIST to volume measurements, thresholds became −65 %; 73 %.

In our case, we were interested in defining thresholds applicable to relative changes and we assumed that such thresholds can be derived from the variability of repeated measurements. According to QIBA’s analysis, our study is based on a 15 % standard deviation of repeated assessments. Considering such a standard deviation, our mathematical model gives a theoretical set of threshold as −35 %; +55 %.

These values of thresholds are found consistent with simulations and with repeated measurements of real clinical data.

To be noted that, unlike RECIST thresholds, the set of threshold we defined for volume incorporates only metrological variabilities.

This conceptual difference can be the origin of the differences we found in comparing RECIST thresholds versus our extrapolation to effective diameter.

A factor that could partly explain such discrepancy is the different strategy chosen to design WHO criteria [30], and consequently RECIST criteria. In 1981 WHO criteria assessed thresholds that were designed to classify between patient’s responses. In our case, our threshold aim at classify significant tumour change not knowing the performance at classifying patients responses.

We identified several limitations to this study. First, the 15 % standard deviation that we considered could be re-evaluated. Considering a greater precision, for example with standard deviations of 5 and 10 %, the theoretical thresholds become −13 %; 15 % and −25 %; 32 % respectively. This observation put in evidence that our approach is particularly sensitive to the choice of the standard deviation.

The images we used to confirm hypothesis did not fully conform to QIBA compliance requirements with regard, for instance, to signal-to-noise ratio or slice thickness. We did not evaluate the consequences of such deviations.

Second, we assumed that volume measurements followed a normal distribution even though some authors suggest a lognormal distribution [9, 27]. Hein and collaborators affirmed that distribution of errors did not follow the pattern for normal distribution but they did not suggest clear justifications [31]. The log transformation of our data gave same rejection probability and, hypothesizing either type of distribution, returned very similar results showing the robustness of our model.

A third limitation may lie in the assumption of proportionality between mean and standard deviation, even if it has also been assumed by other authors [9, 26]. This relationship between mean and standard deviation partially justified the use of relative volume difference to quantify volume measurement variability.

We observed that the assumptions related to the distribution of errors and to constant coefficient of variation were not strictly established, nevertheless our simulations indicate that our model is robust regarding these assumptions. For sake of simplicity, the present investigation suggests keeping these simple assumptions.

This study was designed to apply to a broad types of pulmonary tumours; the goal was to model the most generic set of response threshold. For that goal, we show that, using only simple first order statistics, the model allows for inferring confidence interval or response thresholds. Another approach would have consisted in addressing specific pulmonary tumours or imaging data, for such very specific context our method, due to its theoretical basis, is particularly flexible because requiring only to test limited statistical assumptions. The model can also be used to optimise thresholds for specific image analysis technologies (e.g., segmentation systems) involving given level of automation or tooling.

The development of a practical method able to draw sets of decisional thresholds also opens the door to a panel of new investigations. These investigations would aim at answering questions like: does the same set of thresholds apply in the same way to diverse lesions across the different organs, and what are the most appropriate target lesions that can be reliably monitored? In the future, we could consider by-organ specialised sets of response thresholds that would lead to the design of more complete and efficient response evaluation criteria.

As in other fields of drug development, it can be expected that designing more specific criteria will improve the power of clinical studies.

## Conclusion

To perform reliable quantification of radiological changes of patient, response thresholds and/or confidence intervals must be provided along with the evaluation of the imaging biomarker.

In order to propose a set of response threshold applicable to the monitoring of advanced pulmonary cancer on CT, we implemented and validated a mathematical model based on well-established reliability analysis in the field.

While the response thresholds we proposed are based on metrological considerations only, this work is however a basis allowing the use of tumour volumetry thus contributing to the decision making, both in the care of individual patients and in the management of clinical trials.

## Appendix

### *p*-value of the test

The *p*-value is calculated as the lowest probability for which we can still reject the null hypothesis for a given set of observations.-value. The null hypothesis here is *X*_*2*_/*X*_1_ *= 1.* For a given observed ratio *X*_*2*_/*X*_1_, the quantile *t* of standard normal distribution is calculated from the equation:

1 $$ \frac{X_2}{X_1}=\frac{1-t\;\rho {c}_1{c}_2\pm t\;\sqrt{c_2^2-2\rho {c}_1{c}_2+{c}_1^2-{t}^2{c}_1^2{c}_2^2\left(1-{\rho}^2\right)}}{1-{t}^2{c}_2^2} $$

The *p*-value is deduced from the calculation of *t*. If the *p*-value is equal or smaller than the significance level, the null hypothesis is rejected.

### Statistical power of the test

The power of a statistical test is the probability that it correctly rejects the null hypothesis (H0) when it is false. The quantile *t'* of standard normal distribution was calculated assuming the alternative hypothesis of a given ratio *X*_*2*_/*X*_1_ from the equation:

2 $$ \begin{array}{l}\frac{X_2}{X_1}\;\frac{1-{t^{\prime}}^2\;\rho {c}_1{c}_2\pm {t}^{\prime}\sqrt{c_2^2-2\rho {c}_1{c}_2}+{c}_1^2-{t^{\prime}}^2{c}_1^2{c}_2^2\left(1-{\rho}^2\right)}{1-{t^{\prime}}^2{c}_2^2}\\ {}=\frac{1-{t}^2\rho {c}_1{c}_2\pm t\;\sqrt{c_2^2-2\rho {c}_1{c}_2+{c}_1^2-{t}^2{c}_1^2{c}_2^2\left(1-{\rho}^2\right)}}{1-{t}^2{c}_2^2}\end{array} $$

with t the upper α/2 quantile of normal distribution.

The power is then deduced from the value of *t'*.

Thus, from any given ratio *X*_*2*_/*X*_1_, calculation of *p*-value and power could be calculated.
